# Supplementary material for: Implementing routine collection of EQ-5D-5L in a breast cancer outpatient clinic
Source: PLoS One. 2024 Aug 27;19(8):e0307225. doi: 10.1371/journal.pone.0307225 (PMC11349211; doi:10.1371/journal.pone.0307225)
Supplement: S1 Table — (PDF) [file pone.0307225.s001.pdf]

Reasons for being “Unsure”

- “A bit time consuming but if I could be shown the benefit is strong that might make me reconsider.”
- “Depending on how I feel that day.”
- “Depends on how I am feeling.”
- “Depends on how much time I have.”
- “Don’t want to dwell/focus on this more than I already have to.”
- “I am at the clinic every month; feel it would be repetitive.”
- “I sometimes find it annoying to fill out the survey every time I come to the clinic. I usually feel absolutely fine and its the last thing I want to do when I come to the clinic.”
- “If someone helps me answer the questions I will do it.”
- “It depends on the time and symptoms.”
- “Live far.”
- “Not comfortable.”
- “Not sure of length.”
- “Sometimes I get here late for appointments.”
- “Taking too much time.”
- “It seems useless.”
- “Time.”
- “Time consuming not sure if there would be worthwhile outcome.”

- 25 participants gave no reason for their answer.

Reasons for answering “Very unlikely”

- “I don't feel that my treatment is always the reason for the way I'm feeling. Also, the questionnaire seems to be geared for patients receiving chemo.”
- “Feel too exposed doing it and find the volunteers were speaking too loudly and I was not comfortable.”
- “I don’t have any issues so it would be a waste of time.”
- “No time.”
- “Similar to symptom checker.”
- “Time constraints.”
- “Time consuming and would prefer to discuss with a person.”
- “Too many questions.”

- 10 participants gave no reason for their answer.

Reasons for answering “Definitely not”

- “I am healthy.”
- “I don't feel I need to answer the questions because I feel the same every time. Any difference I tell the doctor; questionnaires and doctor ask the same questions.”
- “I don't know why.”
- “I don't think you ask the right questions.”
- “I trust my doctor.”
- “It is not easy for me to come to clinic; arthritis patient.”
- “It takes time away from my appointment.”

- 3 participants gave no reason for their answer.
