# Supplementary material for: Implementing routine collection of EQ-5D-5L in a breast cancer outpatient clinic
Source: PLoS One. 2024 Aug 27;19(8):e0307225. doi: 10.1371/journal.pone.0307225 (PMC11349211; doi:10.1371/journal.pone.0307225)
Supplement: S2 Table — (PDF) [file pone.0307225.s002.pdf]

| Characteristics | **Willing** N=252; 70% | **Unsure/ Unwilling** N= 70; 22% | **Unadjusted OR (95%CI)** | **p-value** |
|---|---|---|---|---|
| **Age** | | | | 0.787 |
| < 45 years (ref.) | 29; 76% | 9; 24% | - | |
| 45-64 years | 152; 80% | 39; 20% | 1.21 (0.53-2.76) | |
| ≥ 65 years | 71; 76% | 22; 24% | 1.00 (0.41-2.43) | |
| **Charlson Comorbidity Index** | | | | 0.025 |
| 0 (ref.) | | | | |
| 1-2 | 179; 81% | 41; 19% | - | |
| ≥ 3 | 64; 75% | 21; 25% | 0.70 (0.38-1.27) | |
| | 9; 53% | 8; 47% | 0.26 (0.09-0.71) | |
| **Education** | | | | 0.004 |
| ≤ Grade 8 (ref.) | 3; 27% | 8; 73% | - | |
| High school | 45; 80% | 11; 20% | 10.91 (2.48-48.00) | |
| College/ university | 142; 78% | 40; 22% | 9.47 (2.40-37.35) | |
| Postgraduate / professional | 62; 85% | 11; 15% | 15.03 (3.44-65.61) | |
| **Primary Language** | | | | 0.423 |
| English (ref.) | 163; 80% | 42; 20% | - | |
| Other | 87; 76% | 28; 24% | 0.80 (0.47-1.38) | |
| **Breast Cancer State** | | | | 0.383 |
| State 1 | 61; 79% | 16; 21% | 1.51 (0.73-3.15) | |
| State 2 | 3; 60% | 2; 40% | 0.60 (0.09-3.80) | |
| State 3 | 85; 82% | 19; 18% | 1.77 (0.89-3.55) | |
| State 4 | 45; 82% | 10; 18% | 1.78 (0.77-4.13) | |
| State 5 (ref.) | 58; 72% | 23; 28% | - | |
| **EQ-5D-5L Mobility** | | | | 0.028 |
| No problems (ref) | 179; 82% | 40; 18% | - | |
| Problems | 73; 71% | 30; 29% | 0.54 (0.32-0.94) | |
| **EQ-5D-Self-Care** | | | | 0.086 |
| No problems (ref) | 219;80% | 55; 20% | - | |
| Problems | 33; 69% | 15; 31% | 0.55 (0.28-1.09) | |
| **EQ-5D-5L Usual Activities** | | | | 0.549 |
| No problems (ref) | 129; 80% | 33; 20% | - | |
| Problems | 123; 77% | 37; 23% | 0.85 (0.50-1.45) | |
| **EQ-5D-5L Pain / Discomfort** | | | | 0.884 |
| No problems (ref) | | | | |
| Problems | 96; 79% | 26; 21% | - | |
| | 156; 78% | 44; 22% | 0.96 (0.56-1.66) | |
| **EQ-5D-5L Anxiety/ Depression** | | | | 0.432 |
| No problems (ref) | 102; 76% | 32; 24% | - | |
| Problems | 150; 80% | 38; 20% | 1.24 (0.73-2.11) | |
