# Supplementary material for: Implementing routine collection of EQ-5D-5L in a breast cancer outpatient clinic
Source: PLoS One. 2024 Aug 27;19(8):e0307225. doi: 10.1371/journal.pone.0307225 (PMC11349211; doi:10.1371/journal.pone.0307225)
Supplement: S3 Table — (PDF) [file pone.0307225.s003.pdf]

**Characteristics**	**Willing** N=252; 78%	**Unsure / Unwilling** N= 70; 22%	**Adjusted OR (95% CI)**	**p-value**
**Age**				0.577
< 45 years	29; 76%	9; 24%	reference	
45-64 years	152; 80%	39; 20%	1.57 (0.64-3.86)	
≥ 65 years	71; 76%	22; 24%	1.66 (0.60-4.61)	
**Charlson Comorbidity Index**				0.064
0	179; 81%	41; 19%	reference	
1-2	64; 75%	21; 25%	0.80 (0.41-1.55)	
≥ 3	9; 53%	8; 47%	0.27 (0.09-0.81)	
**Education**				0.008
Grade 8 education or less	3; 27%	8; 73%	reference	
Some of completed High school	45;80%	11; 20%	11.09 (2.36-52.18)	
Some of completed college or university	142; 78%	40; 22%	9.65 (2.25-41.52)	
Some of completed postgraduate / professional	62; 85%	11; 15%	14.77 (3.10-70.33)	
**Primary Language**				0.499
English	163; 80%	42; 20%	reference	
Other	87; 76%	28; 24%	0.81 (0.45-1.48)	
**Breast Cancer State**				0.506
State 1	61; 79%	16; 21%	1.71 (0.74-3.91)	
State 2	3; 60%	2; 40%	0.64 (0.09-4.32)	
State 3	85; 82%	19; 18%	1.75 (0.83-3.70)	
State 4	45; 82%	10; 18%	1.57 (0.62-4.00)	
State 5	58; 72%	23; 28%	Reference	
**EQ-5D-5L Mobility**				0.332
No problems (ref)	179; 82%	40; 18%	reference	
Problems	73; 71%	30; 29%	0.69 (0.32-1.47)	
**EQ-5D-5L Self-Care**				0.338
No problems (ref)	219;80%	55; 20%	reference	
Problems	33; 69%	15; 31%	0.66 (0.28-1.55)	
**EQ-5D-5L Usual Activities**				0.577
No problems (ref)	129; 80%	33; 20%	reference	
Problems	123; 77%	37; 23%	1.25 (0.58-2.69)	
**EQ-5D-5L Pain / Discomfort**				0.840
No problems (ref)	96; 79%	26; 21%	reference	
Problems	156; 78%	44; 22%	1.08 (0.51-2.28)	
**EQ-5D-5L Anxiety/ Depression**				0.246
No problems (ref)	102; 76%	32; 24%	reference	
Problems	150; 80%	38; 20%	1.47 (0.77-2.80)	
