# Supplementary material for: Implementing routine collection of EQ-5D-5L in a breast cancer outpatient clinic
Source: PLoS One. 2024 Aug 27;19(8):e0307225. doi: 10.1371/journal.pone.0307225 (PMC11349211; doi:10.1371/journal.pone.0307225)
Supplement: Attachment — Submitted filename: Response to reviewers.pdf [file pone.0307225.s005.pdf]

Sofia Torres

sofia.cidtorres@mail.utoronto.ca

17 November 2023

Elisa Ambrosi, PhD, Academic Editor

Plos One

Dear Dr. Ambrosi and reviewers,

On behalf of my colleagues, as corresponding author, I would like to thank the editor and external reviewers for the thoughtful review of our manuscript entitled "*Implementing routine collection of EQ-5D-5L in a breast cancer outpatient clinic.*".

The paper was reviewed, and the only additional comment by reviewer 1 addressed. Reviewer 2 had no further comments. All the following alterations are tracked and explained on the reviewed manuscript:

Reviewer 1:

Comment: "I've observed that the authors have supplied some information for the my previous comment (see below) in the submission system, but it would be great to include the review contents in the manuscript, either in the literature review section or the discussion section. Please revise the manuscript to include them.

There are similar studies, please review them and describe the similiarties and differences between the existing studies and the present study: https://hqlo.biomedcentral.com/articles/10.1186/s12955-022-02047-0 https://www.thieme-connect.com/products/ejournals/html/10.1055/s-0043-116223 https://www.sciencedirect.com/science/article/pii/S2212109918300487"

Answer to comment: As explained in our previous answer, our discussion already included several studies we considered relevant for the discussion (lines 375 to 386). The studies mentioned by the reviewer have significant differences in terms of objectives, methodology, population included and setting, compared with our study. Those differences make them sufficiently dissimilar that we do not feel that we need to include them either in the literature review or the discussion. Nevertheless, we added a paragraph acknowledging those differences in the Introduction section (lines 85 to 90 of the revised manuscript).

I hope to have answered all the concerns raised by the reviewers. Please feel free to contact me if you have any questions or require additional information. My fellow authors and I thank you for considering our manuscript for publication in *Plos One*.

Kind regards,

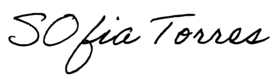

Sofia Torres
