# Supplementary material for: Implementing routine collection of EQ-5D-5L in a breast cancer outpatient clinic
Source: PLoS One. 2024 Aug 27;19(8):e0307225. doi: 10.1371/journal.pone.0307225 (PMC11349211; doi:10.1371/journal.pone.0307225)
Supplement: Attachment — Submitted filename: Response to reviewers.pdf [file pone.0307225.s006.pdf]

Sofia Torres

sofia.cidtorres@mail.utoronto.ca

6 February 2024

Elisa Ambrosi, PhD, Academic Editor

Plos One

Dear Dr. Ambrosi and reviewers,

On behalf of my colleagues, as corresponding author, I would like to thank the editor and external reviewers for the thoughtful review of our manuscript entitled "*Implementing routine collection of EQ-5D-5L in a breast cancer outpatient clinic*".

The paper was reviewed. Reviewer 1 had no further comments and considered that all comments were addressed. Reviewer 2 had additional comments which we have addressed in this review. All the following alterations are tracked and explained on the reviewed manuscript:

Reviewer 2:

Comment: "Thank you for clarifying the focus of the data collection - namely to inform HTA decision-making. At one level your study demonstrates a degree of feasibility in administering EQ-5D in a clinic setting. However, around 30% of eligible patients declined to participate (hence likely skewed results, favouring the better educated, less sick patients). Additionally, as noted in the discussion, of those who did participate, there was a "surprisingly" high rate of patients who reported no problem on ALL dimensions (ie by definition -being in full health). SO ... whilst demonstrating practical FEASIBILITY the study also highlights the issue of what might be called "legitimacy". In essence. does EQ-5D "work" in this clinic setting and/or for this condition. Concerns

around these issues are only briefly alluded to in the Discussion and one might be forgiven for not confronting them here. HOWEVER, the Conclusion (and Abstract) claim that the study indicates that it is feasible, ... to obtain up-to-date population-based HRQOL data that could be used to inform drug and health technology reimbursement and decision making in Ontario. Frankly, this statement is at odds with the evidence presented in this study. It is sufficiently wide of the mark that even at this late stage I would urge the authors to consider addressing it. In all other respects the manuscript is fine - of course there can be differences based on style, ethos, beliefs etc .... but this conclusion risks being cited as demonstrating MORE THAN PRACTICAL FEASIBILITY. Given the uncertainty regarding the performance of EQ-5D and patient refusal rates, it would be the height of folly to unconditionally consider this as the basis for obtaining (patient) HrQoL data to inform high-level decision-making."

Answer to comment: Thank you for the additional comments. We removed the following sentences respectively from the abstract and the conclusions: "in order to obtain up-to-date population-based HRQOL data that could be used to inform drug and health technology reimbursement and decision making in Ontario." (lines 53-54) and, "in order to obtain up-to-date population-based HRQOL data that could be used to inform drug and health technology reimbursement and decision making in Ontario." (lines 448-450).

We made the following changes in the Abstract (lines 52-56): "Tablet-based collection of EQ-5D-5L in the context of routine clinical practice proved to be feasible. However, many patients declined study participation or reported being in full health, raising concerns about whether this method of collecting EQ-5D adequately represents the health status of all breast cancer patients."

In the Manuscript we added the following sentence to the Discussion (lines 387-388): "or whether our study population was representative of all breast cancer patients.". The

Conclusions were also updated (lines 446-452): “In conclusion, our study demonstrated that tablet-based collection of EQ-5D-5L in the context of routine clinical practice was feasible for breast cancer patients. EQ-5D-5L can be easily administered, in addition to the Symptom Screening and Functional Status questionnaires. However, many patients declined study participation or reported being in full health, raising concerns about whether this method of collecting EQ-5D adequately represents the health status of all breast cancer patients.”

I hope to have answered all the concerns raised by the reviewers. Please feel free to contact me if you have any questions or require additional information. My fellow authors and I thank you for considering our manuscript for publication in *Plos One*.

Kind regards,

Sofia Torres

Sofia Torres
